# A Glance at the Nuclear Envelope Spectrin Repeat Protein 3

**DOI:** 10.1155/2019/1651805

**Published:** 2019-11-20

**Authors:** Liwei Liao, Rongmei Qu, Jun Ouang, Jingxing Dai

**Affiliations:** ^1^First School of Clinical Medicine, Southern Medical University, Guangzhou, China; ^2^Guangdong Provincial Key Laboratory of Medical Biomechanics, Department of Anatomy, School of Basic Medical Science, Southern Medical University, Guangzhou, China

## Abstract

Nuclear envelope spectrin repeat protein 3 (nesprin-3) is an evolutionarily-conserved structural protein, widely-expressed in vertebrate cells. Along with other nesprin family members, nesprin-3 acts as an essential component of the linker of nucleoskeleton and cytoskeleton (LINC) complex. Naturally, nesprin-3 shares many functions with LINC, including the localization of various cellular structures and bridging of the nucleoskeleton and cytoskeleton, observed *in vitro*. When nesprin-3 was knocked down *in vivo*, using zebrafish and mouse models, however, the animals were minimally affected. This paradoxical observation should not limit the physiological importance of nesprin-3, as recently, nesprin-3 has reignited the interest of the research community in studies on cancer cells migration. Moreover, nesprin-3 also plays an active role in certain developmental conditions such as adipogenesis and spermatogenesis, although more studies are needed. Meanwhile, the various protein binding partners of nesprin-3 should also be emphasized, as they are necessary for maintaining the structure of nesprin-3 and enabling it to carry out its various physiological and pathological functions. Nesprin-3 promises to further our understanding of these complex cellular events. Therefore, this review will focus on nesprin-3, examining it from a genetic, structural, and functional perspective. The final part of the review will in turn address the limitations of existing research and the future perspectives for the study of nesprin-3.

## 1. Introduction

Nuclear envelope spectrin repeat protein 3 (nesprin-3), also known as Klarsicht, ANC-1, or Syne Homology (KASH) domain-containing protein 3, was first discovered and reported by Kevin Wilhelmsen et al. in 2005 [1]. Nesprin-3 is recognized as a highly conserved protein, located in the outer nuclear membrane (ONM), and widely distributed throughout various vertebrate tissues. Nesprin-3 is the third member of the nesprin family, which form essential components of the nucleoskeleton and cytoskeleton (LINC) complex. The LINC complex plays a vital role in connecting the nucleoskeleton and the cytoskeleton, localization and migration of nuclei [2], maintenance of cell morphology and tension [3], and the transmission of mechanical force [4]. It has been reported that the mutation, destruction, or overexpression of LINC components can affect a chain of cellular events, leading to developmental disorders or dysfunction of the skeletal muscle, heart, peripheral nerves, bones, adipose tissue, and brain, resulting in various diseases affecting the tissue(s) or organ(s) implicated [5–7]. Since the discovery of nesprin-3, extensive research has been performed looking at the genetic coding and expression, protein structure, and functional characteristics of this vital component of the LINC complex. This brief review describes the latest research into the structure and function of nesprin-3.

## 2. The Nesprin-3 Gene

The gene encoding nesprin-3 is called SYNE3, but is also known as C14orf139, C14orf49, or LINC00341. SYNE3, which is 58.2 kb in size and consists of 18 exons and 17 introns, is located on chromosome 14q32.13.

## 3. The Expression and Structure of Nesprin-3

### 3.1. The Distribution and Expression of Nesprin-3

Nesprin-3 is widely distributed throughout the various tissues of vertebrates, but its expression levels vary depending on the tissue type. Referring to the *Homo sapiens* GEO (Gene Expression Omnibus) NCBI (National Center for Biotechnology Information) database, nesprin-3 (Gene ID: 161176) is most highly expressed in adipose tissue (RPKM 4.3) and bone marrow (RPKM 3.9). In contrast, the pancreas and the salivary gland express the lowest levels of nesprin-3. Additionally, a study looking at identifying brain-cell-specific nuclear proteins also discovered that nesprin-3 is significantly enriched in nonneuronal nuclei [8]. The unequal distribution of nesprin-3 may imply that this protein performs different functions depending on its expression level in the tissue or cell type.

### 3.2. The Common Structural Features of Nesprins

The nesprin proteins are essentially made up of three parts: a C-terminal KASH domain, a series of spectrin repeat (SR) domains and an actin-binding domain (ABD). Nesprins are typical KASH proteins, with their C-termini inserting into the nuclear membrane bilayer. At the nuclear envelope, three independent hook-like KASH peptides bind to the SUN (Sad1p, UNC-84) domains of the SUN homotrimer, located at the inner nuclear membrane (INM) [9]. In this way, a SUN-KASH heterohexamer complex is formed, which serves as a basic structural unit of the LINC complex. Furthermore, the N terminus of the SUN protein stretches into the nucleoplasm, where it can interact with the nuclear lamina protein A (lamin A) [9]. The formation of the SUN-KASH complex is an essential step in enabling the nesprins to bind to the ONM [10]. For example, if the KASH domain is damaged or the SUN protein is knocked out, nesprin-3*α* will enter the endoplasmic reticulum (ER) instead, localizing away from the nuclear membrane, and co-localizing with the plectin protein [10]. Lamin attachment also helps with correct localization, albeit in a limited capacity, as SUN can still locate the INM even in the absence of lamin A or lamin B [11].

**Figure 1:**
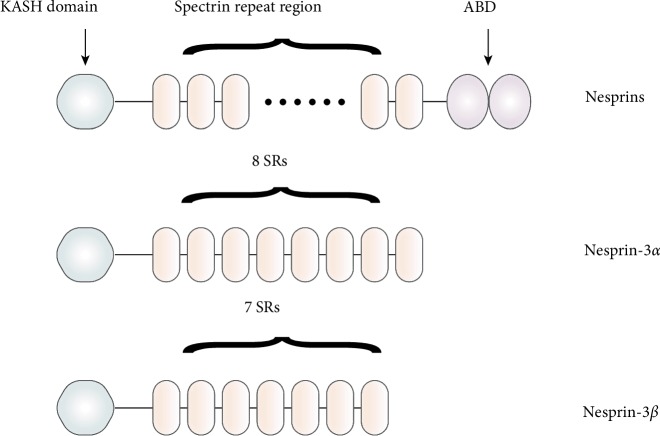
The structure of nesprin-3. Nesprin-3 consists of a KASH domain and a series of SRs. Different from nesprin-1/2, nesprin-3 is lack of ABD. Nesprin-3 encodes two isoforms, nesprin-3*α* and nesprin-3*β*. The main structural difference is that nesprin-3*α* contains 8 SRs, while nesprin-3*β* only contains 7.

In contrast to the conserved C-terminal of the nesprin family, the nesprin N-terminus is highly diverse, owing to the variable number of SR motifs and the presence or absence of the ABD. This structural flexibility allows the nesprins to connect with actin filaments, microtubules, and intermediate filaments of the cytoskeleton. The SR itself is a 106–122 amino acid segment, comprising three *α*-helices. The SRs are arranged in series and joined by an inner link domain of a helix, to form either an SR domain or a rod domain. In nesprins, the number of SR copies ranges from 1 to 74. The SR rod domain not only acts as a physical spacer to determine the distance between the cytoskeleton and the nuclear membrane [12], it can also serve as a binding domain that enables nesprins to interact with other proteins [13]. ABD, also known as the calponin homology domain (CHD), is essentially composed of two tandem calmodulin homologous domains, CH1 and CH2. While CH1 can directly join to actin filaments, CH2 cannot do so unless it combines with CH1. The resulting CH1-CH2 binding domain binds actin filaments with higher affinity than CH1 alone. In addition, mutations affecting CH2 can also change the affinity of CH1 [14]. ABD is located near the nesprin amino terminus and contains four *α*-helices of 11–18 residues in length, with typically four parallel and three interspersed short helices (Figure 1).

### 3.3. The Unique Structural Features of Nesprin-3

Nesprin-3 only contains a KASH domain and a spectrin repeat (SR) region composed of 7 or 8 spectrin repeats. Nesprin-3 encodes two isoforms, nesprin-3*α* and nesprin-3*β*. While, nesprin-3*α* contains 8 SRs, nesprin-3*β* has an additional transcription starting site, resulting in the absence of the first SR, and only containing SRs 2–8. Based on these, nesprin-3 is naturally structurally similar to other nesprins, but also has its own special traits.

Firstly, the way that nesprin-3 connects to the cytoskeleton is different from the binding methods used by nesprins 1 and 2. The ABD, present in both nesprins 1 and 2, is absent from nesprin-3, preventing nesprin-3 from directly binding to the cytoskeleton. Instead, the N-terminus of nesprin-3 can indirectly connect to the cytoskeleton when it binds another ABD protein.

Secondly, nesprin-3 is much smaller than nesprin-1 or nesprin-2. As well as not having an ABD domain, nesprin-3 only contains 7 or 8 SRs, while nesprin-1 has 74 SRs and nesprin-2 has 56. The molecular weight of nesprin-1 is 976 kDa, while nesprin-2 weighs 764 kDa [15]. In contrast, nesprin-3 only weighs 110 kDa. The main splice isoforms of nesprin-3 are nesprin-3*α* and nesprin-3*β* [1].

The C-terminal localization of nesprin-3 dictates its distribution on the ONM, as is the case for other nesprins. However, it should be noted that nesprin-3 is not always localized to the ONM and can occasionally be observed in nonnuclear-membrane regions such as the rough ER, a phenomenon especially common in tissues where the expression level of nesprin-3 is highest.

In addition, recent research has revealed that certain differentiation events are also associated with changes in nesprin-3 localization. For instance, nesprin-3 was shown to migrate to the cytoplasm of preadipocytes in the early stages of adipogenesis. Interestingly, adipose tissue is associated with the highest level of nesprin-3 expression. The predicted new location of nesprin-3 is likely to be the ER, which is coincidentally also the site of lipid droplet formation. However, the specific mechanisms involved remain to be determined [1]. Moreover, the migration of nesprin-3 during adipogenic differentiation appears to be a dynamic process. In the middle stages of adipogenesis, nesprin-3 gradually relocates once more to the nuclear membrane [17, 18], but is observed surrounding lipid droplets in the ER, at the end of adipogenesis [18]. Also during spermatogenesis, nesprin-3 leaves the ONM and relocates to the sperm's anterior pole, forming a complex with SUN1 at this new site [19]. These observations may indicate that the changes in the localization of nesprin-3 may be related to some specific processes, such as in this instance, differentiation. The cytoskeleton can participate in and promote cellular differentiation by regulating cell morphology [20], and the ability to interact with various binding partners may modulate the association between nesprin-3 and the cytoskeleton. Overall, many questions concerning the expression, localization, diversity of binding partners and perhaps the differential functions of nesprin-3 in different cell types, require further attention.

## 4. The Function of Nesprin-3

The crosstalk between nesprin-3 and its binding partners forms an important structural basis for the various functions of nesprin-3 and represents a key area of research. As a component of the LINC complex, nesprin-3 naturally exhibits some basic functions attributed to the LINC. More specifically, nesprin-3 bridges the nucleoskeleton and the cytoskeleton, and additionally participates in the localization and migration of intracellular structures.

### 4.1. The Physiological Function of Nesprin-3

#### 4.1.1. Nesprin-3 Connects the Nucleoskeleton and the Cytoskeleton

Nesprin-3 helps to form and maintain a complete LINC complex. As previously mentioned, the LINC is formed following a series of SUN-KASH binding events at the nuclear membrane, which includes the binding of nesprin-3 to the SUN proteins. In such a complex, the SUNs interact with lamin A to connect with the nucleoskeleton inside the nuclear membrane, while nesprin-3 links to the cytoskeleton outside of the nuclear membrane. To attach to the cytoskeleton, nesprin-3 first needs to bind to an ABD of another protein. Though nesprin-3 exists in two isoforms, nesprin-3*α* and nesprin-3*β*, only nesprin-3*α* can attach to the cytoskeleton. Nesprin-3*α* can indirectly bind to the intermediate filaments of the cytoskeleton via the plectin protein [20], but it can also do so by linking the nuclear membrane to the microtubules via an interaction with Microtubule-actin cross-linking factor (MACF) [15] and Bullous pemphigoid antigen 1 (BPAG1). BPAG1 is a kind of Cytoskeletal linker protein which is localized in cell membrane, nuclear membrane [22], ER and even cytoskeleton. BPAG1 has eight isoforms, but only BPAG1-*α* contains an ABD domain [22], making it possible to interact with nesprin-3*α* [24]. However, nesprin-3*β* has a weak affinity for ABD proteins as it lacks the first SR, which is critical for this interaction.

#### 4.1.2. Nesprin-3 Is Associated with the Localization and Migration of Intracellular Structures

The LINC complex not only physically links the cytoskeleton to the nucleoskeleton, but also provides them with a functionally active interface. In many cases, the LINC complex instructs the cytoskeleton to steer directional nuclear movement [25, 26]. Consequently, nesprin-3 also influences the localization and migration of nuclear and other intracellular structures. Nesprin-3 does not only bind to proteins in a 1 : 1 ratio but also often interacts with several proteins simultaneously to form a functional structural network, used to accomplish specific functions in unison. Here are some representative examples.

TorsinA (also known as dystonia 1, DYT1) is a type of AAA+ATPase (ATPases associated with various cellular activities) expressed in many human tissues. TorsinA and torsinB [27] can both interact with the nesprin KASH domain at the nuclear membrane. This interaction was believed to help nesprin-3 localize to the ONM, as evidenced by the fact that nesprin-3 relocates from the nuclear membrane to the ER when torsinA is knocked out [27]. In addition, a torsinA-nesprin-3-plectin-vimentin complex was observed in fibroblasts, thereby constructing an interconnected mesh between the nucleus and the plasma membrane [27]. Although the phenotype of fibroblasts remained normal when torsinA was knocked out, nuclear polarization and cell migration were both delayed in a wound closure assay. This observation indicates that the interaction between torsinA and nesprin-3 plays a role in nuclear and cellular movement. Additionally, the migration of torsinA in cells also deserves attention. TorsinA is primarily localized to the ER, while a small amount is found at the INM. However, the localization of TorsinA is dynamic, shuttling between the ER and the INM. Transmembrane protein lamina-associated polypeptide 1 (LAP1) [27] (also known as TOR1AIP1) and LULL1 (luminal domain like LAP1) [29] in ER (also Called TOR1AIP2 or NET9) are associated with the transport of torsinA between the ER and the INM.

More specifically, the up-regulation of LULL1 expression can induce more torsinA migration from the ER to the INM, resulting in the displacement of nuclear membrane proteins such as SUN2, nestrin-2G and nesprin-3, while leaving nuclear pores and SUN1 unchanged [29]. It can be inferred that torsinA is vital for maintaining the structural integrity of the LINC complex and the ONM localization of nesprin-3. We can also speculate that torsinA may be one of the pathways employed to regulate the positioning of nesprin-3, to regulate its distribution between the nuclear membrane and the ER.

The role of nesprin-3 in cellular and nuclear migration has been demonstrated. Nesprin-3 not only regulates cell morphology, but also mediates the polarization of the cell and its centrosomes [29]. When cells migrate in a 3D matrix, an actomyosin-vimentin-nesprin-3 complex is formed [31]. This complex is vital for the cells' ability to generate the high-pressure lobopodium, a temporary cellular protrusion that is uniquely formed during 3D cell migration. The role of nesprin-3 within the actomyosin-vimentin-nesprin-3 complex is that of a mediator. Nesprin-3 transfers the force of contraction from the actomyosin to the nucleus, dragging the nucleus forward and pressurizing the front of the cell. Due to this cellular pressure difference, a lobopodium forms at the cell's anterior. On knocking out nesprin-3, the corresponding intracellular pressure was significantly equalized, causing the lobopodium to transform into lamellipodia. Since 2D cell migration mainly relies on the formation of lamellipodia [32], it is reasonable to assume that nesprin-3 is only essential for 3D migration through the matrix.

Nesprin-3 can also interact with other nesprin family members such as nesprin-1 [33] and nesprin-2 [34], as they both have an ABD domain. This connection has little impact on either the nesprin-3-plectin complex formation or the nesprin-1-actin interaction. Instead, the nesprins, together with the cytoskeletal proteins form a perinuclear network that maintains the morphology of the nucleus and mediates nuclear movement. This supports the existence of a protein network, which is reminiscent of the spectrin network found in the cytoskeleton of erythrocytes [35].

Besides rescuing nesprin-3, torsinA can also regulate the migration of nuclei by adjusting the LINC complex [36]. Another study has revealed an additional connection between nesprin-3 and nuclear localization, by showing that sets of LINC proteins, including nesprin-3, were downregulated in cells stimulated by microgravity, promoting cell apoptosis by altering nuclear positioning [37].

#### 4.1.3. Nesprin-3 Knockout Experiments In Vivo

Since it has been proved *in vitro* that nepsrin-3 engages in many important cellular events, especially those involving the LINC complex or the cytoskeleton, relevant experiments *in vivo* have also been carried out. However, when nesprin-3 was knocked out in zebrafish and mice, both nesprin-3-dificient zebrafish [38] and mice [39] exhibited normal morphology and even produced fertile offspring. These results were unexpected, as no apparent disorders were observed, in total contrast to the cases involving the knockout of nesprin-1 or nesprin-2. Deletion of nesprin-1 or nesprin-2 can result in cardiomyopathy [40], and knockout of all nesprin-2 isoforms even caused early embryonic death [41]. To this surprising result, there are several possible explanations:

(1) There might be an adaptive response to the absence of nesprin-3 in cells. For example, cells may strengthen the roles of other nesprin members and promote the association between intermediate filaments and actin filaments.
(2) The experimental duration may be too short to observe the potentially chronic impact of knocking out nesprin-3.
(3) The experiments and tests on nesprin-3-knockout animals are inevitably incomplete, so perhaps some abnormalities, requiring specific stimuli, were missed. For instance, researchers are curious about whether there are any abnormal cardiac responses to stress in nesprin-3-dificient mice, as the absence of nesprin-3 *in vitro* causes defects in cell migration and morphology [42].
(4) Even if nesprin-3 participates in many significant physiological functions, the effect of nesprin-3 knockout may not necessarily be proportional to the variety of its functions.
(5) This result might suggest that nesprin-3 is not so indispensable under normal physiological conditions.

### 4.2. The Pathological Function of Nesprin-3

The *in vivo* findings do not necessarily imply that nesprin-3 has no physiological value. On the contrary, under certain pathological conditions, especially in cancer and the genetic disease DYT1 dystonia, the actions of nesprin-3 become more apparent.

To this end, nesprin-3 has been attracting increasing attention in the field of Oncology. At the genetic level, one study found that the amplification or deep deletion of SYNE3 is associated with the development of human epithelial-type tumors, based on the oncoprint of epithelial cancer. This evidence suggests that altering the copy number of SYNE3 may represent a new strategy for the prevention and control of certain carcinomas [43]. The actomyosin-vimentin-nesprin-3 complex plays a vital role in the formation of the high-pressure lobopodium, which drives fibroblasts to migrate in a 3D matrix. This mechanism could be exploited to inhibit cancer migration [44], especially as promising new research has found that this same mechanism in also employed by migrating fibrosarcoma cells [45].

The role of vimentin in the mechanism of normal cell migration [46] and tumor transplantation [47] has also been previously proven, and nesprin-3 may act as an accessory component in this setting. Sperm-associated Antigen 4 (SPAG4), sometimes referred to as SUN4, is highly expressed in the testis and the pancreas, but rarely detected in other tissues. However, in recent years, SPAG4 was found greatly upregulated in a wide range of neoplastic tissues, eventually, becoming a new marker for lung cancer [47]. Because of its SUN domain, SPAG4 can also interact with nesprin-3 [49]. The study revealed that this interaction promotes the development of lung cancer, and a reduction in the migration of lung cancer cells can be observed when nesprin-3 or SPAG4 are knocked out [49].

**Figure 2:**
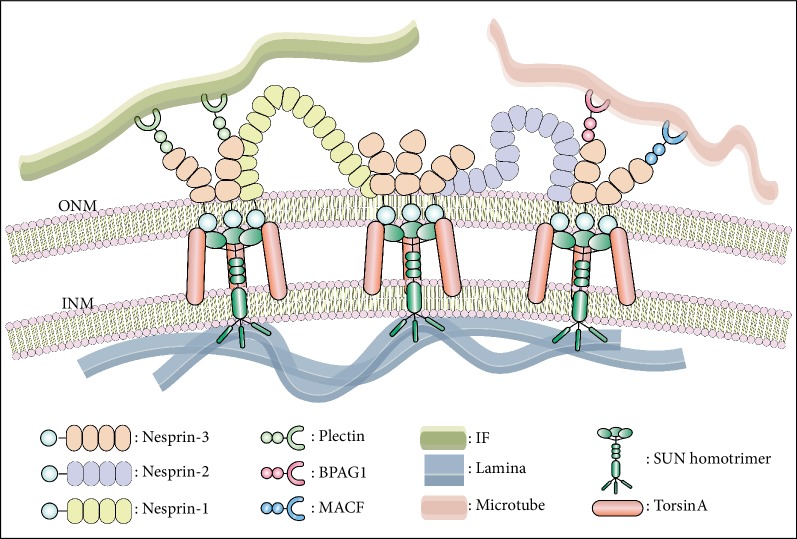
Nesprin-3 and its binding partners. Nesprin-3 is located on ONM. At nuclear membrane, it can interact with SUN proteins located on INM, meanwhile torsinA also bind with this combination to firm it. Inside the nuclear membrane, SUN proteins can interact with the nuclear lamin A, which connecting nucleoskeleton to LINC complex. Outside the nuclear membrane, nesprin-3 can link to cytoskeleton though indirectly. Plectin helps nesprin-3 connect to IF, while BPAG1 and MACF attach nesprin-3 to microtubes. Moreover, nesprin-3 can interact with other nesprins, like nesprin-1 and nesprin-2.

DYT1 dystonia is a neurological disease caused by a dominant mutation. It is one of the most common forms of early-onset isolated dystonia, which arises from the deletion of a glutamate residue within torsinA. This torsinA mutation can affect glycoprotein homeostasis, the ER stress response, lipid metabolism, calcium homeostasis, synaptic function and neurodevelopment, and cause abnormal transport of nuclear proteins to the cytoplasm [51]. In addition, dysfunctional torsinA causes the cells to partially lose their regulation of the LINC complex, providing a plausible mechanism for the onset of DYT1 dystonia. Nesprin-3 is thus also likely to have an involvement in this disease [51]. Indeed, the fibroblasts of DYT1 dystonia sufferers have been shown to accumulate nesprin-3 in globular structures within the ER lumen [27], although the underlying mechanisms have not been clearly elucidated (Figure 2 and Table 1).

## 5. Conclusions and Perspectives

In this review article, nesprin-3 has been examined from three different perspectives: genetic, structural, and functional. As a newly discovered nesprin protein, studies on nesprin-3 are relatively limited. Nesprin-3 is involved in the formation of the LINC complex, the bridging of the cytoskeleton and the nucleoskeleton, and the localization of intracellular structures. More recently, nesprin-3 was found to function in various physiological and pathological processes, thus attracting more and more attention from researchers. However, there are numerous limitations of the existing nesprin-3 research, which are summarized in the following points:

(1) Although nesprin-3 exists in two isoforms, most of the existing studies have only focused on nesprin-3*α* or have not deliberately distinguished the two isoforms. Therefore, it is not clear how nesprin-3*β* and nesprin-3*β* are different or similar. Nesprin-3*α* should not be excluded from further research.
(2) Nesprin-3 is a member of the nesprin family, and all the nesprins are components of the LINC complex. Classic examples of cytoskeletal research can be re-visited, to explore the role of nesprin-3, especially relating to work involving the cytoplasmic intermediate filament network. Nesprin-3 is the only nesprin that can connect the nucleoskeleton to the intermediate filament network. Moreover, alongside the large nesprins 1 and 2, the molecular weight of nesprin-3 is comparatively small. Is it possible that these features endow nesprin-3 with some unique functions? In addition to performing research on previously-developed study models, there is also a need to study nesprin-3 under new, special conditions.
(3) The non-nuclear localization of nesprin-3 is also noteworthy. Nesprin-3 has been shown to migrate from the nuclear membrane to the cytoplasm, in a rare and unique phenomenon. For instance, nesprin-3 is observed to diffuse into cytoplasm at the early stage of adipogenesis, arranging in a loose circle. While during spermatogenesis, nesprin-3 is detected at the anterior pole of cells when forming the sperm head. However, it remains unknown whether and how nesprin-3 participates in these developmental processes, signaling the need for further exploration.
(4) Immunofluorescence is the most commonly-used method for measuring the intracellular localization of nesprin-3. However, as immunofluorescence relies heavily on the effective interaction between antibodies and their antigens, there are limitations to this approach. Nesprin-3 forms complexes with multiple proteins, which may obscure the antibody binding site and interfere with accurate detection [27].
(5) Although nesprin-3 contributes to many important cellular functions, the results from *in vivo* knockout experiments proved disappointing. In agreement with the results obtained from experiments on nesprin-3-dificient zebrafish and mice, mentioned earlier, no diseases caused by the deletion, mutation or overexpression of nesprin-3 have been reported. This unexpected phenomenon needs more analysis, to address whether the body employs mechanisms to compensate for the loss of nesprin-3.

Although no direct link between the loss of nesprin-3 function and specific diseases has been identified in healthy individuals, researchers have recently started paying attention to how nesprin-3 may be involved in certain pathologies, especially in DYT1 dystonia and cancer. And while few pathological mechanisms have been clearly defined, further *in vivo* experiments using pathological animal models should be planned to address the role of this mysterious protein.

## Figures and Tables

**Table 1:** Binding partners of nesprin-3 and their functions.

Protein	Binding requirements	Functions	References
SUN proteins: SUN1, SUN2, SUN3, SUN4 (SPAG4) and SUN5 (SPAGL4)	The last four amino acids (PPPT) of the nesprin-3 KASH domain are required.	SUN proteins can interact with lamin A, thus nesprin–SUN can bridge nucleoskeleton and cytoskeleton.	[3, 9, 10, 15, 19, 49, 50]
Cytoskeletal linker proteins: plectin, BPAG1 and MACF	Two residues in the first spectrin repeat of nesprin-3*α* are essential for this interaction.	Can link NE to IFs or microtubules; plectin can also cross-link IFs with F-actin; BPAG1 and MACF can connect nesprin-3 with microtubule and actin cytoskeleton.	[1, 15]
AAA + proteins: torsinA and torsinB	KASH domain of nesprin-3 is the binding domain.	Keep the integrity of LINC complexes; migration of torsinA from ER to NE can relocate nesprin-3.	[15, 25, 29]
Other nesprins: nesprin-1 and nesprin-2	Second spectrin in nesprin-3 is needed.	Form a interacted network covering the outer nuclear membrane, which maintains nuclear morphology and mediates nuclear movement.	[33, 34]
